# Molecular Study on Twin Cohort with Discordant Birth Weight

**DOI:** 10.3390/antiox12071370

**Published:** 2023-06-30

**Authors:** Payal Chakraborty, Hajnalka Orvos, Edit Hermesz

**Affiliations:** 1 Department of Biochemistry and Molecular Biology, Faculty of Science and Informatics, University of Szeged, P.O. Box 533, H-6701 Szeged, Hungary; payal.chakraborty@jisuniversity.ac.in; 2 Department of Pharmaceutical Technology, JIS University, 81, Nilgunj Road, Kolkata 700109, India; 3 Department of Obstetrics and Gynaecology, Faculty of Medicine, University of Szeged, Semmelweis u. 1, H-6725 Szeged, Hungary; orvosh@obgyn.szote.u-szeged.hu

**Keywords:** birth-weight discordance, free radicals, hypoxia, nitric oxide synthases, oxidative stress, peroxynitrite, red blood cells, umbilical cord vessels

## Abstract

The increased rate of twinning has pointed out newer challenges in clinical practices related to gestational complications, intrauterine growth restriction, perinatal mortality, and comorbidities. As a twin pregnancy progresses, the increased demand for oxygen supply can easily disrupt the redox homeostasis balance and further impose a greater challenge for the developing fetuses. A substantial birth-weight difference acts as an indicator of a deficit in oxygenation or blood flow to one of the fetuses, which might be related to a low bioavailable nitric oxide level. Therefore, in this study, we focused on networks involved in the adjustment of oxygen supply, like the activation of inducible and endothelial nitric oxide synthase (NOS3) along with free radical and lipid peroxide formation in mature twin pairs with high birth-weight differences. The selected parameters were followed by immunofluorescence staining, fluorescence-activated cell sorting analysis, and biochemical measurements in the umbilical cord vessels and fetal red blood cells. Based on our data set, it is clear that the lower-weight siblings are markedly exposed to persistent intrauterine hypoxic conditions, which are connected to a decreased level in NOS3 activation. Furthermore, the increased level of peroxynitrite aggravates lipid peroxidation and induces morphological and functional damage and loss in redox homeostasis.

## 1. Introduction

Multiple/twin pregnancies have significantly increased in the last few decades, which can be partly traced back to the applied expansion of assisted reproduction techniques and a steep surge in the average maternal age during the first pregnancy [1,2]. The increased rate of twinning has pointed out newer challenges in clinical practices related to gestational complications [3]. The appropriate fetal development critically depends on the efficiency of the fetoplacental circulation through the umbilical cord (UC) vessels. The activation of molecular parameters involved in the regulation of the oxygen supply of the developing fetus, like endothelial nitric oxide synthase (NOS3), its phosphorylation status at the serine1177 residue (pNOS3), and the upregulation of the inducible nitric oxide synthase (NOS2), is crucial. The UC vein carries oxygenated blood, while arteries carry the deoxygenated blood in the vascular system. With any UC disorder, structural and/or functional alterations would reflect the *in-utero* condition and can be directly or indirectly connected to intrauterine hypoxia, impaired blood flow to the developing fetus, and retardation in fetal growth. Since UC vessels lack innervation, the regulation of nitric oxide (NO) production by NOS3 expression and activation in the vessels counts as determining factors in the control of blood flow and maintenance of the vascular tone [4,5]. Alteration in the NOS3 expression and activation and/or NO degradation due to the reactive oxygen species (ROS) may serve as markers for an unfavorable cardiovascular condition. A low amount of bioavailable NO in the vessel walls can lead to the formation of endothelial dysfunction (ED), which may cause cardiovascular complications during fetal and post-natal life [6,7,8]. As an alternative to the NO-producing pathway, the upregulation of inducible nitric oxide synthase (NOS2) expression may serve as a rescue mechanism to increase the NO level in vascular circulation [9,10,11,12,13]. Apart from the upregulation of NOS2, hypoxic condition and the deficiency of bioavailable NO can be sensed by healthy fetal red blood cells (RBCs). Therefore, under stressful conditions, RBCs themselves are able to synthesize NOS3 (RBC-NOS3) and can serve as an emergency NO-producing entity [14,15,16].

Per the study design, neonates are categorized based on their birth weights as appropriate, large, and small for the gestational age. In addition, especially for twin/multiple pregnancies, significant birth-weight differences may occur between the siblings. Based on the consensus laid down by the American College of Obstetricians and Gynecologists (2016), the threshold value of birth-weight discordance was considered to be between 20–25% difference in the actual weight between the twin siblings.

In our previous publications, we followed the NO-producing pathways in singletons and birth weight–non-discordant, mature and pre-mature twin pairs [13,17]. We presented significant evidence for the altered NOS3 expression and its activation capacity in the UC vessels depending on the gestational age and birth weights of the twin neonates. Additionally, we presented evidence that besides the vascular walls, the RBC-NOS3-NO pathway also gets activated during the pre-term pregnancy status. However, as the pregnancy progresses (37–39 weeks), there arises a higher demand for NO production because of the increasing hypoxic conditions with an elevated NO degradation rate due to ROS. In mature twin populations, we have seen that there lies a significant impairment in the NO-producing capacity via the NOS3 pathway, along with an increase in birth weight [13,17].

ED refers to several pathological conditions and impairment in endothelium-dependent vasorelaxation due to the loss of NO bioactivity [18,19]. In recent years, special attention has been paid to the investigation of NO-mediated signaling pathways not only in the vessel endothelium but also in cooperation with the RBCs. Here, we point out the relationship between the birth weight and UC endothelium NOS3-, NOS2-, and the circulating RBC-NO-producing capacity to further support their importance in *in-utero* fetal development. Moreover, a deeper knowledge and understanding of such complementary relationships between these complex systems will allow them to emerge as early biomarkers associated with the risk of cardiovascular events.

## 2. Materials and Methods

### 2.1. Clinical Samples

Umbilical cord samples along with UC arterial blood were collected at the Department of Obstetrics and Gynecology at the University of Szeged, Hungary, in the time period of 2014–2022. Samples from informed volunteers with twin pregnancies were handled according to the Declaration of Helsinki. The study protocol was approved by the Institutional Ethics Committee (16/2014 and 20/2016). In the requisition process, we excluded clinical factors like (i) maternal age below 18 years; (ii) gestational age less than 37 weeks; (iii) gestational diabetes, infection, and inflammatory conditions or disorders such as cardiovascular diseases; (iv) complications or difficulty during delivery, (v) malformations or evidence of genetic disorders; and (vi) neonates from mothers addicted to alcohol or with smoking habits. Under any circumstances, the nutritional status of the women during pregnancy was kept satisfactory, and no cases of malnutrition had been reported. Here, in total, we considered 18 pairs of mature, birth weight–discordant siblings (with a minimum of 25% birth-weight difference) and grouped them according to their birth-weight and gestational-percentile ranges; likewise, high weight–discordant (D-Hwt) and low weight–discordant (D-Lwt) twin siblings have birth-weight ranges of 2800–3500 g and 2100–2600 g, respectively, and gestational percentiles of ~75–95 percent and ~10–50 percent, respectively. The average timing between the twin delivery was 1–10 min, depending on the mode of delivery (vaginal deliveries, on average, had a maximum difference of about 10 min; in the case of cesarean section, it was only 1–3 min). In detail, the clinical parameters of the above-mentioned data sets are demonstrated in Table S1 (Supplementary Material).

### 2.2. Immunohistochemistry in the Umbilical Cord Sections

Sample processing: Small segments of the umbilical cords of 2–3 cm in length were immediately fixed in 4% (*w*/*v*) paraformaldehyde in 0.05 M phosphate buffer (PB) upon arrival and cryopreserved with 30% (*w*/*v*) sucrose in PB added with 0.1% (*v*/*w*) Na-azide. Samples were kept at 4 °C until the further process of embedding. Fixed UC segments were embedded in Tissue-Tek^®^ O.C.T.™ (4583) from Sakura Finetek Europe (Alphen aan den Rijn, The Netherlands) for the purpose of cryosectioning slice thicknesses of 16 µm. Finally, the UC sections were mounted on Superfrost™ ultra plus^®^ microscope slides (J3800AMNZ) from Thermo Fisher Scientific (Waltham, MA, USA) and stored at −80 °C until the following steps [20].

Immunohistochemistry on the specimen samples: The slides with the UC sections were thawed, dried at room temperature (RT), and permeabilized with 0.1% Triton X-100 for 20 min, which was followed by the blocking of a nonspecific binding using 4% (*w*/*v*) bovine serum albumin (BSA) and 5% (*v*/*v*) normal goat serum (NGS) in PB. Using the process of immunohistochemistry technique, UC sections were immunolabelled with the primary antibodies at 4 °C overnight. The slides, after consecutive washing, were further incubated with the secondary antibodies, goat anti-mouse Alexa^®^ 647- and/or goat anti-rabbit Alexa^®^ 488-conjugated in 1:2000 dilution, in the dark for 2 h at RT. Finally, for the nuclei staining, slides were washed with 0.05 M PB 2–3 times and counterstained with 4′,6-diamidino-2-phenylindole (D9542) from Sigma-Aldrich (St. Louis, MO, USA), using a concentration of 1 µg/cm^3^, in the dark for at least 5 min [21].

Visualizing using Fluorescence Microscope: To check and analyze under an epifluorescence microscope (Nikon Eclipse 80i, 100× and 50× immersion objective; Nikon Zeiss Microscopy GmbH, Jena, Germany), the immunolabelled slides were semi-dried and mounted using Antifading, Bright Mount/Plus aqueous mounting medium (ab103748) from Abcam (Cambridge, UK). Images were captured using a QImaging RETIGA 4000R camera with Capture Pro 6.0 software (QImaging, Surrey, BC, Canada). The acquired images with 100× or 50× magnification were semi-quantitatively analyzed using the ImageJ^®^ 1.50i software (National Institutes of Health, Bethesda, MD, USA).

### 2.3. Immunocytochemistry and Fluorescence-Activated Cell Sorting (FACS) on the Fetal RBCs

The whole blood was immediately processed upon arrival for the RBC fraction isolation; the whole blood was subjected to centrifugation at 200× *g* for 10 min at an optimum temperature of 22 °C, and the lower 2/3 of the RBC populations were collected. The purity of the isolated RBC was checked by staining with the RBC-specific marker anti-Glycophorin A (CD235a) from Thermo Fisher Scientific (Waltham, MA, USA), where the sample purity was >95% [21]. The freshly isolated RBCs were fixed using 4% (*w*/*v*) paraformaldehyde in 0.05 M PB at 4 °C in the neutral pH for 1 h. Next, using 0.1% Triton X-100 for 30 min, the RBCs were permeabilized at RT. After permeabilization, the RBCs were blocked for nonspecific antibody binding for 1 h in PB containing 4% (*w*/*v*) BSA and 5% (*v*/*v*) NGS. After blocking, the RBCs were immunolabelled with the primary antibodies at 4 °C overnight. This was followed by consecutive washing and incubation with the goat anti-mouse Alexa^®^ 647 and goat anti-rabbit Alexa^®^ 488-conjugated secondary antibodies for 2 h at RT (see Table S2 for the detailed list of antibodies). In the final step, the RBCs were thoroughly washed and prepared for quantification through FACS analysis (FACS, BD FACSCalibur™; BD Biosciences, Franklin Lakes, NJ, USA) using the software FlowJo™ (FlowJo™ Software for Windows Version 10; Ashland, OR, USA).

### 2.4. Determination of Peroxynitrite (ONOO^−^) Level

An isolated RBC fraction was divided into aliquots and stored at −20 °C until further processing for biochemical measurements. To determine the ONOO^−^ levels of the RBC populations, spectrophotometric measurements were performed at 302 nm using a GENESYS 10S UV-Vis spectrophotometer. Hemolyzed samples were mixed with 1 M NaOH solution in a ratio of 1:250, and the increase in absorbance was followed until it reached a stable equilibrium. Then, the samples were mixed with 100 mM PB (pH = 7.4) in 1:250 ratios. On this neutral pH, the ONOO^−^ decomposed, and a decrease in the absorbance was observed until the equilibrium point [22].

The ONOO^−^ concentration was calculated as a result of the different absorbance at the two distinct pH values according to the Lambert–Beer law (Ɛ_ONOO−_ = 1670 M^−1^ cm^−1^). The final results were calculated relative to the protein concentration (μmol/mg protein).

### 2.5. Blood Smear Image Processing and Data Analysis

The total blood originating from the siblings with substantial birth-weight differences (n = 18 pairs) was used upon arrival for smear preparation. Smears were immunolabelled for anti-NOS3/Alexa^®^ 647-conjugated antibodies and mounted in Antifading, Bright Mount/Plus aqueous mounting medium (Abcam ab103748, Cambridge, UK). Blood smears were examined under the epifluorescence microscope (Nikon Eclipse 80i, 100× immersion objective; Nikon Zeiss Microscopy GmbH, Jena, Germany) with a QImaging RETIGA 4000R camera using Capture Pro 6.0 software (QImaging, Surrey, BC, Canada). Smear images were processed and analyzed using the scientific software MatLab. Different phenotypic variants were calculated using the automated bio-image analysis tool CellProfiler™ (available at http://cellprofiler.org and accessed on 15 November 2018) [23].

### 2.6. Statistical Analysis

Statistical significance was accepted at values ** *p* ≤ 0.01 and *** *p* ≤ 0.001. All calculations were performed through one-way analysis of variance (ANOVA) and complied with the Newman–Keuls multiple comparison test using the GraphPad Prism Statistical Software version 6.0.

## 3. Results

### 3.1. Expression of Endothelial and Inducible NOS in the Birth Weight–Discordant Populations

Molecular parameters of UC arteries and veins originating from twin pairs with high birth-weight differences were compared. First, sections of the vessels were double immunolabelled for NOS3/pNOS3 and subjected to Image J© (1.50i) evaluation.

**Figure 1:**
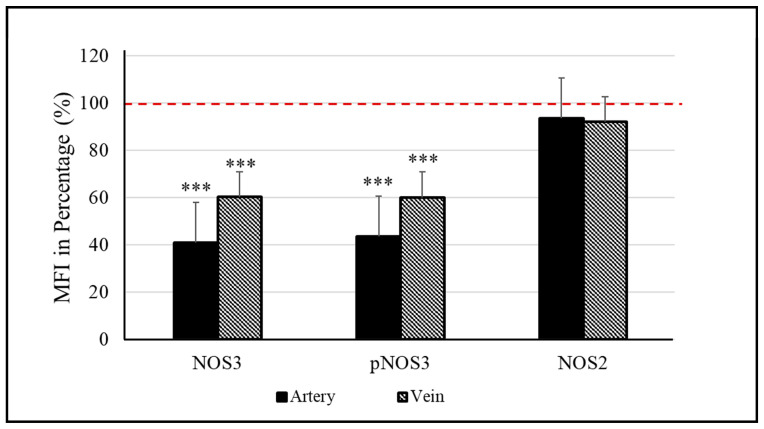
Endothelial (NOS3) and inducible nitric oxide synthase (NOS2) levels in the umbilical cord vessels. Quantifying the mean fluorescence intensity (MFI) in percentage determines the level of NOS3 and its phosphorylated status at Ser1177 residue (pNOS3) along with the expression of NOS2 of the low-weight twin neonates (D-Lwt), where the values of their high-weight siblings (D-Hwt) were taken as 100% (red dotted line) (n = 18 pairs). Statistical significance was accepted at *** *p* < 0.001 based on one-way ANOVA using the Newman–Keuls multiple comparison test. Statistical analysis was performed on the measured MFI data set.

Considering the high- versus low-weight siblings, there is a significant difference between their NOS3 expressions/activations, both in the vessels. In the arteries, a ~60% lower value was measured for both the NOS3 and pNOS3 intensities. The vein was also affected, but the level of alteration was remarkably smaller, ~40% in both the NOS3 expression and the Ser1177 phosphorylation intensity in the lower-weight siblings (Figure 1, Figures S1 and S3). In the case of impaired NOS3 expression/activation, an increased NOS2 level could serve as a rescue mechanism to increase the bioavailable NO level. However, in both vessels, the NOS2 level was found to be similar between the siblings (Figure 1, Figures S2 and S3); there was only a 5–8% difference in the measured intensity values.

Comparing the mature, birth weight–discordant and non-discordant populations, in the case of high-weight neonates, the measured parameters were comparable for both the vessels and RBCs (Figure S4). The discordant population was “even in a better condition” in terms of NOS3 expression and activation; the efficacy increase was about 20–40% in both the veins and the RBCs. However, the values of the low-weight siblings were far below those of their age-matched, low birth-weight, non-discordant twin pairs, especially in the case of arteries and RBCs. In the arteries, there were ~40% and 30% differences in the NOS3 expression and its activation, respectively, and neither the NOS2 activity in the vessels nor the circulating RBC populations were able to compensate for the impaired NO production level (Figure S5).

### 3.2. NOS3 Expression and Activation Level in the Red Blood Cell of the Birth Weight–Discordant Study Populations

**Figure 2:**
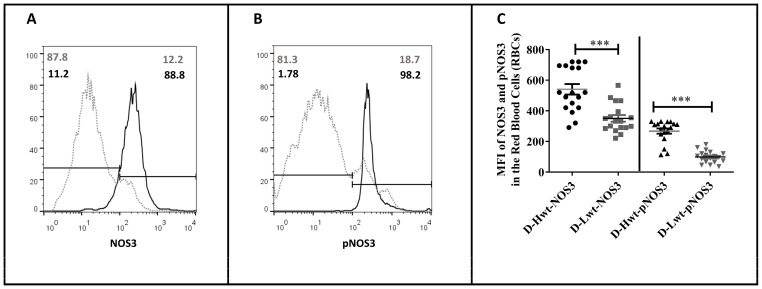
Fluorescence-activated cell sorting analysis of the endothelial nitric oxide synthase (NOS3) expression and its activation status on the immunolabelled fetal red blood cells (RBCs). Panels (**A**,**B**) show representative histogram plots indicating NOS3 and pNOS3 intensity levels in the mature, discordant high-weight (D-Hwt—black line) and low-weight (D-Lwt—gray dots) twin siblings. According to the blank sample, an arbitrary borderline at 10^2^ was considered dividing the total RBC population into basal and high intensity levels. Graph (**C**) quantifies the mean fluorescence intensity (MFI) values of the total NOS3 expression and its phosphorylation status on the immunolabelled UC arterial RBCs., as derived from both the Hwt and Lwt mature discordant (n = 18 pairs) twin siblings. The statistical significance was accepted at *** *p* < 0.001 based on one-way ANOVA using the Newman–Keuls multiple comparison test.

NOS3 protein level and its activation by the phosphorylation at the Serine1177 position were immunostained for NOS3/Glycophorin and pNOS3/Glycophorin and analyzed using FACS (Figure 2). Representative histograms indicate that, in the low-weight neonates, both the NOS3 expression and activation (pNOS3) were far behind the higher-weight siblings’ values (an arbitrary borderline at 10^2^ was considered along the x-axis dividing the total RBC population into basal- and high-intensity groups). In the higher-weight neonates, ~89% and 98% of the Glycophorin-positive cells showed high NOS3 and pNOS3 intensity levels, respectively, whereas, in the case of their smaller siblings, these numbers reached only 12% and 18% (Figure 2A,B). Taking into account the total data set (n = 18), it is clear that there are differences of 35–38% and 55–62% in NOS3 and pNOS3 intensity levels, respectively, between the siblings (Figure 2C).

### 3.3. Membrane Damage Verification in the UC Vessels’ Endothelium and Red Blood Cells; Measurement of 4-Hydroxynonenal Level as a Lipid Peroxidation Marker

**Figure 3:**
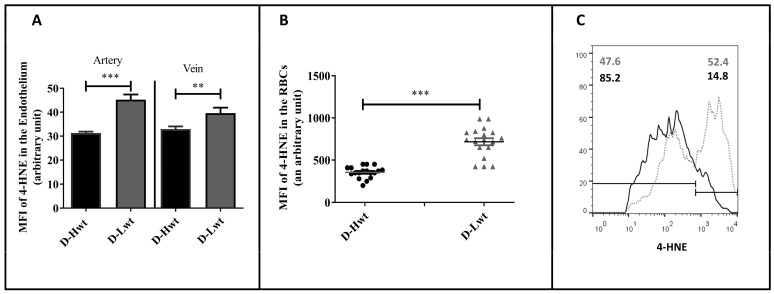
4-hydroxynonenal (4-HNE) level in the immunolabelled umbilical cord (UC) vessels and red blood cells (RBCs) of the mature, birth weight–discordant twin neonates. Graph (**A**,**B**) determines the mean fluorescence intensity (MFI) values to measure the 4-HNE level in the immunolabelled UC vessel sections and the UC arterial RBCs, respectively, between high-weight (D-Hwt -black) and low-weight (D-Lwt—gray) siblings (n = 18 pairs). Panel (**C**) shows representative histograms as measured from the fluorescence-activated cell sorting analysis using anti-4 HNE primary antibodies on the UC arterial RBCs, (D-Hwt—black line) and low-weight (D-Lwt—gray dots). According to the blank sample, an arbitrary borderline at 10^3^ was considered dividing the total RBC population into basal and high intensity levels. All the measured statistical significance was accepted at ** *p* < 0.01 and *** *p* < 0.001 based on one-way ANOVA using the Newman–Keuls multiple comparison test.

During the period of twin pregnancies, there exists a high intra-uterine hypoxic environment. Subsequently, there occurs an excessive production of ROS, which induces the formation of oxygenated α, β-unsaturated aldehydes, such as 4-hydroxynonenal (4-HNE). The lipid peroxidation level was followed by immunolabelling the aldehyde-protein adduct using an anti-4-HNE antibody. In the vessels, though, there were significant differences between the high and low birth-weight siblings in the study population (~35–40% of the higher value was indicated in the arteries, and a 15% rise occurred in the vein) (Figure 3A); the major differences were measured in the circulating RBCs. The intensity level (MFI value) of 4-HNE doubled in the smaller-weight siblings (Figure 3B), and ~53% of the RBCs showed extremely high intensity values (i.e., above 10^3^) based on the FACS analyses data set (Figure 3C).

### 3.4. Peroxynitrite (ONOO^−^) Accumulation in Red Blood Cell Populations

**Figure 4:**
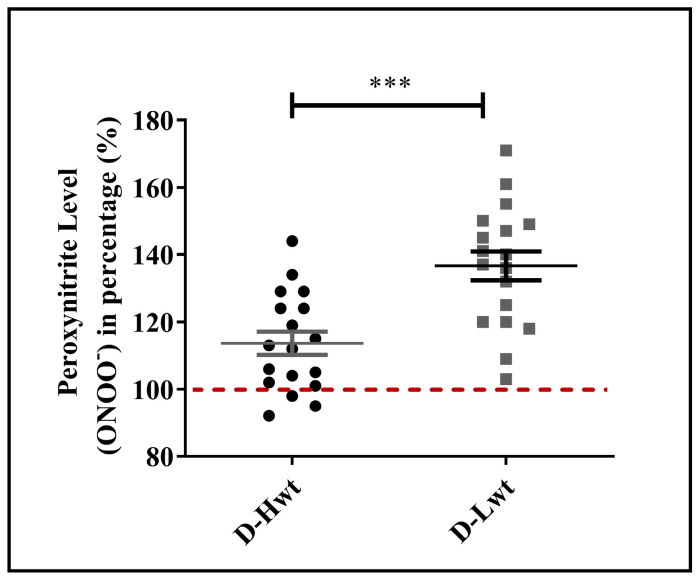
Peroxynitrite level in the mature, birth weight–discordant twin population. Peroxynitrite levels of RBC populations derived from mature (D-Hwt—black and D-Lwt—gray), birth weight–discordant siblings (n = 18 pairs). Values were expressed in percentages of age- and weight-matched, mature, birth weight–non-discordant twin neonates, taken as control (100%). Statistical significance was accepted at *** *p* < 0.001 by one-way ANOVA using the Newman–Keuls multiple comparison test.

One of the major culprits for the elevated level of lipid peroxidation is the increased formation of ONOO^−^ via the spontaneous reaction between superoxide anion (O_2_^•−^) and NO. More than 50% of the RBC samples derived from the low-weight population showed a highly increased level of ONOO^−^ (40–60% differences between siblings), while the high-weight siblings’ ONOO^−^ levels were quite comparable to their age- and weight-matched, birth weight–non-discordant twin neonates (Figure 4 and Figure S6).

### 3.5. Accumulation of Morphological Variants in the Birth Weight–Discordant Red Blood Cell Populations

**Figure 5:**
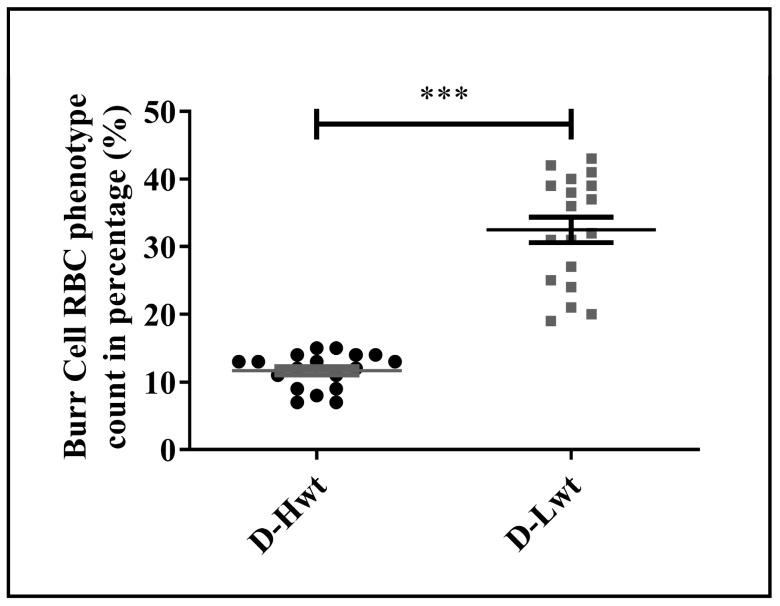
Distribution of the phenotypical variations in the fetal red blood cell population. Frequency of the Burr cell count was obtained as percentage with respect to healthy biconcave-shaped cells taken as control, where the total cell count of approximately ~17,000 cells was considered from the (n = 18 pairs) mature, birth weight–discordant twin siblings. Statistical significance was accepted at *** *p* < 0.001 by one-way ANOVA using the Newman–Keuls multiple comparison test.

As a further consequence of the substantial stress condition, significant alterations in the phenotypical variants were detected, and as a result, RBCs were losing their characteristic biconcave disk-shaped structures. Altogether, ~17,000 cells were counted from the n = 18 pairs of siblings and analyzed using the bio-image analysis tool Cell Profiler™. In the Lwt siblings, the Burr cell population was found to be ~30% of the total RBC population (Figure 5 and Figure S7), and even besides that, a formation of the Rouleaux phenotype was also detectable within ~4–5%.

## 4. Discussion

Substantial birth-weight differences between the twin siblings indicate a clinical condition of insufficient oxygenation and reduced blood flow to one of the fetuses. Birth-weight discordance may be well-connected to long-term intrauterine hypoxia and low bioavailable NO levels. In this study, mature, birth weight–discordant twin pairs were analyzed, mostly focusing on the NO-producing capacity. In the vascular system, the bioavailability of NO is crucial for the regulation of redox balance and functionality, most particularly in the case of the UC with no innervation. UC vessel-related complications can be a useful device for the study of various pathophysiological mechanisms related to neonatal cardiovascular disorders. Additionally, impairment in the primary source of NO production via the NOS3 signaling pathway offers an opportunity to have an insight into the potential compensatory mechanisms, like the NOS2 upregulation and the activation of the RBC-NOS3-NO pathway.

First, we checked the values of higher-weight siblings, considered them as a reference, and found that the NOS3 system and its activation status are highly undermined in siblings with low percentile values. In parallel, the NOS2 isoform might play an active role in order to maintain adequate vascular tone since its catalytic activity is 100–1000-fold that of NOS3 [11]. However, in the birth weight–discordant siblings, there were no signs of NOS2 upregulation in any of the vessels. Additionally, in the low–birth weight neonates, the RBC-NOS3 system was also not available to further increase the level of bioavailable NO. Impairment in the RBC-NOS3 system might be associated with an elevated level of ONOO^−^ formation (~20–60% increase). Among the other factors, NOS3 was tightly regulated by its substrate and cofactors availability [24]. The process of NOS3 coupling is the most crucial step toward its activation. So, the oxidative milieu–induced deficiency of cofactors, like tetrahydrobiopterin, is one of the major limitations of NOS3 homodimer formation, resulting in the generation of O_2_^•−^ instead of NO [25,26]. Thus, here, the NOS3 itself becomes a source of free radicals by creating a loop of spontaneous reaction, where the elevated level of O_2_^•−^ scavenges the limited bioavailable NO by forming the deleterious ONOO^−^ entity [27]. In our study, nearly 45% of the lower-weight siblings showed a 30–60% increase in the ONOO^−^ levels. The NOS3-O_2_^•−^ activation phenomenon in the RBCs, especially in the neonatal system, was so far without any example, but currently, endothelial cell lines have shown quite a few supportive results [27,28,29,30,31]. Accumulation of the highly reactive ONOO^−^ is toxic for the cell not only because of its impact on the NO production but also because it can even initiate and aggravate the rate of lipid peroxidation in the cellular membranes, resulting in the excessive accumulation of the aldehyde, 4-HNE. The 4-HNE is highly reactive and can form adducts with nucleic acids [32], phospholipids, and nucleophilic amino acids, such as cysteine, histidine, and lysine residues. Moreover, the 4-HNE cross-linked conjugates propagate lipid peroxidation with subsequent loss in redox homeostasis and lead to pathological disorders [33,34,35,36]. In our data set, it was quite striking that the formation of 4-HNE was exceptionally higher in the case of RBCs derived from the low-weight siblings in comparison to the vascular endothelial cells. Again, the high ONOO^−^ level in the circulating adult RBCs was somehow also responsible for the increase of Arginase1 level both in the RBCs, as well as in the vessel endothelium. The increased level of Arginase1 might play a pivotal role in the formation of ED [37].

In addition to the functional impairment of the RBCs, their morphological changes indicate a massive response to stress exposure in siblings with lower birth weights. There is not much information about the frequency of fetal/neonatal morphological variants of RBCs. Based on Zipursky and coworkers’ study on mature and pre-mature neonatal blood samples, only 43–39% of the RBCs showed regular discoid shapes, while in the healthy adult population, this value reached ~78% [38]. The exact mechanism responsible for the Burr cell formation is unknown, but this type of shape transformation might be associated with the decrease in the concentration of ATP within the cell [39]. The depletion of the ATP pool affects the oxygen affinity of hemoglobin, and the release of oxygen may be less effective from the RBCs with this kind of phenotype [40]. Ruef and Linderkamp published a detailed analysis of the Burr cell population for mature and pre-mature singletons with values of 1.4 and 1.7%, respectively [41]. In our practice, we found that an average of 3% of Burr cell variants was present in the neonates born from healthy single pregnancies. On the other hand, in the case of singletons born to smoking mothers [16], this value has reached 15–18%. Here, appearances of the Burr cell population were connected to the highly elevated level of ONOO^−^ formation and 4-HNE production. In the case of mature or pre-mature twins, we measured ~10–12% of this type of phenotypical variant, with a clear indication of elevated oxidative stress. It has been documented that an increase in the formation of Burr cells population can be well-connected to some pathological events, like that of kidney failure, pyruvate kinase deficiency, and certain liver diseases [42,43,44].

Our data set clearly indicates that there lie significant differences between the arteries and veins. It is always the artery where the impairment in the NO-producing capacity is more prominent, especially in the case of the low-weight siblings. Summarizing this fact with the highly impaired RBC functionality, it seems like the fetoplacental circulation gets remarkably hindered in comparison to the uteroplacental circulation in the lower-weight population.

The guidelines for uncomplicated twin pregnancies and the suggested protocol for a healthy gestational period, as described by the American College of Obstetricians and Gynecologists and the Society for Maternal-Fetal Medicine, respectively, is between 38 and 39 weeks. Despite this, from the clinician’s point of view, a clause often arises whether an earlier timing, even at the late pre-mature period, may be more favorable for neonatal outcome [45,46]. Based on our present and previous studies on twins, we assume that an earlier delivery time can be considered, especially in the case of mature, birth weight–discordant pregnancy. It seems like, in this case, neither the NOS3-NO-producing capacity nor the “built in” rescue mechanisms, like NOS2 and RBC-NOS3, are sufficient enough to support adequate blood flow to the lower-weight fetuses. Contrastingly, in our recent publications on mature and pre-mature, birth weight–non-discordant and pre-mature, birth weight–discordant twin neonates, NOS2 was highly upregulated in the arteries in the case of impaired NOS3 performance, which showcased a compensatory mechanism [13,17]. Similarly, we also demonstrated the pivotal role of fetal RBCs with an export mechanism of NO bioactivity for the lower-weight siblings, regardless of maturity. The RBC populations in all such cases still had the capacity to increase the NO production via upregulation of the RBC-NOS3-NO pathway. This phenomenon suggested an efficient crosstalk mechanism between the RBCs and the impaired vascular system [13,17].

In spite of the major differences between the birth weight–discordant siblings, the lower-weight siblings still come under the category of appropriate for gestational age. Moreover, as examined in our previous study, this weight range is similar to the low-weight, mature, non-discordant twin pairs [13]. Based on the comparison of our data sets (Figure S5), it might be clarified that, in the D-Lwt neonates, the NO production capacity of the arterial vessels, in addition to the RBC populations, remains far behind the weight- and age-matched birth weight–non-discordant twins. This fact further supports our earlier hypothesis that, in the case of the low weight–discordant neonates, it is the fetoplacental circulation that gets hampered and not the uteroplacental one. The UC is fully embryonic in origin, and the vessels in it are considered to be a direct elongation of the vascular system for the developing fetus. This is the reason it is important to highlight that not only the birth weight but also the overall status of the endothelial layer in the UC vessels, along with the circulating fetal RBCs, form the determining factors for the healthy condition of neonates. Our results clearly indicate that the accumulation of strong pro-oxidant entities, like the toxic ONOO^−^, in the circulation can significantly deteriorate the physiological status of the vascular system.

## 5. Conclusions

In the case of the birth weight–discordant siblings, even though their birth weights matched the appropriate weights for gestational age groups, the molecular parameters suggest that the lower-weight siblings are markedly exposed to persistent, intensive intrauterine hypoxic stress conditions. The RBC population of the same may act as a biosensor along with all the detectable phenotypic alterations and functional impairments, which can be taken as blueprints of such pathological conditions. It is quite probable this may have long-term consequences during the *in-utero* fetal development and even in their post-natal/adult lives.

Conclusively, to mitigate the long-term consequences of an extreme hypoxic condition of the vascular system, an earlier timing of delivery may be beneficial for a better clinical outcome of the lower-weight siblings.

## Data Availability

The data will be available upon request.

## Supplementary Materials

The following supporting information can be downloaded from https://www.mdpi.com/article/10.3390/antiox12071370/s1: Figure S1: Epifluorescent images of the umbilical cord artery and vein endothelium with relation to endothelial nitric oxide synthase (NOS3) expression and its activation (pNOS3) pattern; Figure S2: Representation of epifluorescent images of the umbilical cord vessels endothelium immunolabelled for inducible nitric oxide synthase (NOS2); Figure S3: Endothelial (NOS3) and inducible nitric oxide synthase (NOS2) levels in the umbilical cord vessels; Figure S4: Evaluation of the expression pattern of endothelial (NOS3) and inducible nitric oxide synthase (NOS2) and the phosphorylation status of NOS3 (pNOS3) at the Ser1177 residues in between the mature, birth weight–discordant versus non-discordant high-weight twin groups; Figure S5: Evaluation of the expression pattern of endothelial (NOS3) and inducible nitric oxide synthases (NOS2) and the phosphorylation status of NOS3 (pNOS3) at the Ser1177 residues in between the mature, birth weight–discordant versus non-discordant low-weight twin groups; Figure S6: Distribution pattern of the peroxynitrite level between the mature, birth weight–discordant twin siblings’ Figure S7: Representative epifluorescent images of red blood cell variants originated from high-weight (D-Hwt) and low-weight (D-Lwt) siblings; Table S1: Clinical parameters of the study groups and the maternal age; and Table S2: List of the sources and dilutions of primary and secondary antibodies.

## References

[1] Ochoa J.J., Ramirez-Tortosa M.C., Quiles J.L., Palomino N., Robles R., Mataix J., Huertas J.R. (2003). Oxidative stress in erythrocytes from premature and full-term infants during their first 72 h of life. Free Radic. Res..

[2] Umranikar A., Parmar D., Davies S., Fountain S. (2013). Multiple births following in vitro fertilization treatment: Redefining success. Eur. J. Obstet. Gynecol. Reprod. Biol..

[3] Puccio G., Giuffré M., Piccione M., Piro E., Malerba V., Corsello G. (2014). Intrauterine growth pattern and birthweight discordance in twin pregnancies: A retrospective study. Ital. J. Pediatr..

[4] Fox S.B., Khong T.Y. (1990). Lack of innervation of human umbilical cord. An immunohistological and histochemical study. Placenta.

[5] Vanhoutte P.M., Shimokawa H., Feletou M., Tang E.H.C. (2017). Endothelial dysfunction and vascular disease—A 30th anniversary update. Acta Physiol..

[6] Berk B.C., Haendeler J., Sottile J. (2000). Angiotensin II, atherosclerosis, and aortic aneurysms. J. Clin. Investig..

[7] Granger J.P., Alexander B.T., Llinas M.T., Bennett W.A., Khalil R.A. (2001). Pathophysiology of hypertension during preeclampsia linking placental ischemia with endothelial dysfunction. Hypertension.

[8] Nedeljkovic Z.S., Gokce N., Loscalzo J. (2003). Mechanisms of oxidative stress and vascular dysfunction. Postgrad. Med. J..

[9] Selye H. (1955). Stress and disease. Science.

[10] Geng Y.J., Wu Q., Muszynski M., Hansson G.K., Libby P. (1996). Apoptosis of vascular smooth muscle cells induced by in vitro stimulation with interferon-γ, tumor necrosis factor-α, and interleukin-1β. Arter. Thromb. Vasc. Biol..

[11] Farah C., Michel L.Y.M., Balligand J.L. (2018). Nitric oxide signalling in cardiovascular health and disease. Nat. Rev. Cardiol..

[12] Liu X.M., Chapman G.B., Peyton K.J., Schafer A.I., Durante W. (2002). Carbon monoxide inhibits apoptosis in vascular smooth muscle cells. Cardiovasc. Res..

[13] Chakraborty P., Dugmonits K.N., Orvos H., Hermesz E. (2020). Mature twin neonates exhibit oxidative stress via nitric oxide synthase dysfunctionality: A prognostic stress marker in the red blood cells and umbilical cord vessels. Antioxidants.

[14] Cortese-Krott M., Rodriguez-Mateos A., Sansone R., Kuhnle G., Thasian-Sivarajah S., Krenz T., Horn P., Krisp C., Wolters D., Heiss C. (2012). Human red blood cells at work: Identification and visualization of erythrocytic eNOS activity in health and disease. Blood.

[15] Cortese-Krott M.M., Kelm M. (2014). Endothelial nitric oxide synthase in red blood cells: Key to a new erythrocrine function?. Redox Biol..

[16] Dugmonits K.N., Chakraborty P., Hollandi R., Zahorán S., Pankotai-Bodó G., Horváth P., Orvos H., Hermesz E. (2019). Maternal Smoking Highly Affects the Function, Membrane Integrity, and Rheological Properties in Fetal Red Blood Cells. Oxid. Med. Cell. Longev..

[17] Chakraborty P., Khamit A., Hermesz E. (2021). Fetal oxygen supply can be improved by an effective cross-talk between fetal erythrocytes and vascular endothelium. Biochim. Biophys. Acta-Mol. Basis Dis..

[18] Ding H., Triggle C.R. (2005). Endothelial cell dysfunction and the vascular complications associated with type 2 diabetes: Assessing the health of the endothelium. Vasc. Health Risk Manag..

[19] Barthelmes J., Nägele M.P., Ludovici V., Ruschitzka F., Sudano I., Flammer A.J. (2017). Endothelial dysfunction in cardiovascular disease and Flammer syndrome-similarities and differences. EPMA J..

[20] Nishikawa E., Matsumoto T., Isige M., Tsuji T., Mugisima H., Takahashi S. (2016). Comparison of capacities to maintain hematopoietic stem cells among different types of stem cells derived from the placenta and umbilical cord. Regen. Ther..

[21] Chakraborty P., Dugmonits K.N., Végh A.G., Hollandi R., Horváth P., Maléth J., Hegyi P., Németh G., Hermesz E. (2019). Failure in the compensatory mechanism in red blood cells due to sustained smoking during pregnancy. Chem. Biol. Interact..

[22] Huie R.E., Padmaja S. (1993). The reaction of no with superoxide. Free Radic. Res..

[23] Carpenter A.E., Jones T.R., Lamprecht M.R., Clarke C., Kang I.H., Friman O., Guertin D.A., Chang J.H., Lindquist R.A., Moffat J. (2006). CellProfiler: Image analysis software for identifying and quantifying cell phenotypes. Genome. Biol..

[24] Michel T., Feron O. (1997). Nitric oxide synthases: Which, where, how, and why?. J. Clin. Investig..

[25] Förstermann U., Li H. (2011). Therapeutic effect of enhancing endothelial nitric oxide synthase (eNOS) expression and preventing eNOS uncoupling. Br. J. Pharmacol..

[26] Förstermann U., Sessa W.C. (2012). Nitric oxide synthases: Regulation and function. Eur. Heart J..

[27] Cau S.B.A., Carneiro F.S., Tostes R.C. (2012). Differential modulation of nitric oxide synthases in aging: Therapeutic opportunities. Front. Physiol..

[28] Cosentino F., Lüscher T.F. (1999). Tetrahydrobiopterin and endothelial nitric oxide synthase activity. Cardiovasc. Res..

[29] Pritchard K.A., Groszek L., Smalley D.M., Sessa W.C., Wu M., Villalon P., Wolin M.S., Stemerman M.B. (1995). Native low-density lipoprotein increases endothelial cell nitric oxide synthase generation of superoxide anion. Circ. Res..

[30] Landmesser U., Dikalov S., Price S.R., McCann L., Fukai T., Holland S.M., Mitch W.E., Harrison D.G. (2003). Oxidation of tetrahydrobiopterin leads to uncoupling of endothelial cell nitric oxide synthase in hypertension. J. Clin. Investig..

[31] Laursen J.B., Somers M., Kurz S., McCann L., Warnholtz A., Freeman B.A., Tarpey M., Fukai T., Harrison D.G. (2001). Endothelial regulation of vasomotion in apoE-deficient mice: Implications for interactions between peroxynitrite and tetrahydrobiopterin. Circulation.

[32] Nair J., De Flora S., Izzotti A., Bartsch H. (2007). Lipid peroxidation-derived etheno-DNA adducts in human atherosclerotic lesions. Mutat. Res..

[33] Doorn J.A., Petersen D.R. (2003). Covalent adduction of nucleophilic amino acids by 4-hydroxynonenal and 4-oxononenal. Chem. Biol. Interact..

[34] Benedetti A., Comporti M., Esterbauer H. (1980). Identification of 4-hydroxynonenal as a cytotoxic product originating from the peroxidation of liver microsomal lipids. Biochim. Biophys. Acta.

[35] Esterbauer H., Schaur R.J., Zollner H. (1991). Chemistry and biochemistry of 4-hydroxynonenal, malonaldehyde and related aldehydes. Free Radic. Biol. Med..

[36] Chapple S.J., Cheng X., Mann G.E. (2013). Effects of 4-hydroxynonenal on vascular endothelial and smooth muscle cell redox signaling and function in health and disease. Redox Biol..

[37] Mahdi A., Tengbom J., Alvarsson M., Wernly B., Zhou Z., Pernow J. (2020). Red Blood Cell Peroxynitrite Causes Endothelial Dysfunction in Type 2 Diabetes Mellitus via Arginase. Cells.

[38] Zipursky A., Brown E., Palko J., Brown E.J. (1983). The erythrocyte differential count in newborn infants. Am. J. Pediatr. Hematol. Oncol..

[39] Elgsaeter A., Mikkelsen A. (1991). Shapes and shape changes in vitro in normal red blood cells. Biochim. Biophys. Acta.

[40] Chowdhury A., Dasgupta R., Majumder S.K. (2017). Changes in hemoglobin-oxygen affinity with shape variations of red blood cells. J. Biomed. Opt..

[41] Ruef P., Linderkamp O. (1999). Deformability and Geometry of Neonatal Erythrocytes with Irregular Shapes. Pediatr. Res..

[42] Mandal A.K., Taylor C.A., Bell R.D., Hillman N.M., Jarnot M.D., Cunningham J.D., Phillips L.G. (1991). Erythrocyte deformation in ischemic acute tubular necrosis and amelioration by splenectomy in the dog. Lab. Investig..

[43] Turchetti V., De Matteis C., Leoncini F., Trabalzini L., Guerrini M., Forconi S. (1997). Variations of erythrocyte morphology in different pathologies. Clin. Hemorheol. Microcirc..

[44] Suljević D., Mitrašinović-Brulić M., Fočak M. (2023). L-cysteine protective effects against platelet disaggregation and echinocyte occurrence in gentamicin-induced kidney injury. Mol. Cell. Biochem..

[45] Dodd J.M., Crowther C.A., Haslam R.R., Robinson J.S. (2012). Elective birth at 37 weeks of gestation versus standard care for women with an uncomplicated twin pregnancy at term: The Twins Timing of Birth Randomised Trial. BJOG.

[46] Cheong-See F., Schuit E., Arroyo-Manzano D., Khalil A., Barrett J., Joseph K.S., Asztalos E., Hack K., Lewi L., Lim A. (2016). Prospective risk of stillbirth and neonatal complications in twin pregnancies: Systematic review and meta-analysis. BMJ.

