# Supplementary material for: Molecular Study on Twin Cohort with Discordant Birth Weight
Source: Antioxidants (Basel). 2023 Jun 30;12(7):1370. doi: 10.3390/antiox12071370 (PMC10376082; doi:10.3390/antiox12071370)
Supplement: Supplementary file 1 [file antioxidants-12-01370-s001.zip › antioxidants-2439315-supplementary.pdf]

Supplementary materials

Molecular study on twin cohort with discordant birth weight

Payal Chakraborty, Hajnalka Orvos and Edit Hermesz

Table of Contents

Table S1. Clinical parameters of the study groups and the maternal age

Table S2. List of the sources and dilutions of primary and secondary antibodies

Figure S1. Epifluorescent images of the umbilical cord artery and vein endothelium with relation to endothelial nitric oxide synthase (NOS3) expression and its activation (pNOS3) pattern

Figure S2. Representation of epifluorescent images of the umbilical cord vessels endothelium immunolabelled for inducible nitric oxide synthase (NOS2)

Figure S3. Endothelial (NOS3) and inducible nitric oxide synthase (NOS2) levels in the umbilical cord vessels

Figure S4. Evaluation of the expression pattern of endothelial (NOS3) and inducible nitric oxide synthases (NOS2) and the phosphorylation status of NOS3 (pNOS3) at the Ser1177 residues in between the mature birth weight discordant versus non-discordant **high** weight twin groups

Figure S5. Evaluation of the expression pattern of endothelial (NOS3) and inducible nitric oxide synthases (NOS2) and the phosphorylation status of NOS3 (pNOS3) at the Ser1177 residues in between the mature birth weight discordant versus non-discordant **low** weight twin groups

Figure S6. Distribution pattern of the peroxynitrite level between the mature birth weight discordant twin siblings

Figure S7. Representative epifluorescent images of red blood cell variants originated from high (D-Hwt) and low (D-Lwt) birth weight siblings

Table S1. Clinical parameters of the study groups and the maternal age

**Clinical Parameters**	**Full-term Discordant Twin Neonates**
Numbers of Samples (N)	36
Gestational age at delivery (weeks)	37.75 ± 0.44 (37 - 38.2)
Birth weight (kg)	Hwt = 2.95 ± 0.29 (3.45 – 2.49)<br>Lwt = 2.105 ± 0.32 (2.5 – 1.8)
Difference in birth weight (g)	423.33 ± 443.30 (1090 – 630)
Blood sample pH	7.32 ± 0.03 (7.37 – 7.26)
Maternal Age (years)	36.05 ± 3.63 (41 – 30)

Table S2. List of the sources and dilutions of primary and secondary antibodies

Antibody	Host	Dilution	Code	Distributor
anti-Glycophorin A	mouse	1:50	MA5-12484	Thermo Fisher Scientific, Madison, WI, USA
anti-NOS3	mouse	1:100	sc-376751	Santa Cruz Biotechnology Inc., Dallas, TX, USA
anti-pSer1177 NOS3	rabbit	1:100	SAB-4300128	Sigma Aldrich, Saint Louis, Missouri, USA
anti-NOS2	rabbit	1:100	ab3523	Abcam, Cambridge, UK
anti 4-hydroxy-2-nonenal (4-HNE)	mouse	1:100	ab48506	Abcam, Cambridge, UK
goat anti-mouse Alexa®647	mouse	1:2000	ab150115	Abcam, Cambridge, UK
goat anti-rabbit Alexa®488	rabbit	1:2000	ab150077	Abcam, Cambridge, UK
goat anti-mouse Alexa®488	mouse	1:2000	ab150113	Abcam, Cambridge, UK
goat anti-rabbit Alexa®647	rabbit	1:2000	ab150079	Abcam, Cambridge, UK

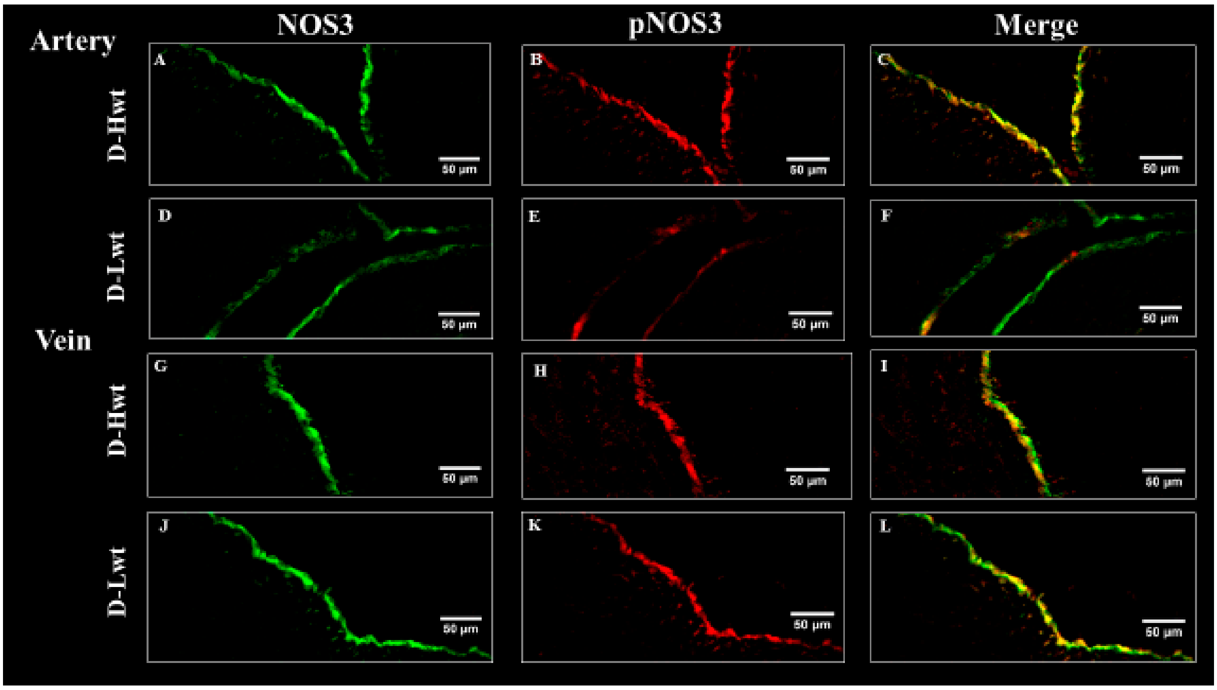

Figure S1. Representative epifluorescent images of the umbilical cord artery and vein endothelium with relation to endothelial nitric oxide synthase (NOS3) expression and its activation (pNOS3) pattern

Representative epifluorescent images of immunolabelled umbilical cord arterial and venous endothelium originated from mature birth weight-discordant siblings (D-Hwt and D-Lwt). Panels (**A, D, G** and **J**) show immunolabelling with mouse primary anti-NOS3 antibody followed by an Alexa Fluor® 488 secondary antibody. Panels (**B, E, H** and **K**) show immunolabelling with a rabbit anti-pSer1177 NOS3 primary antibody followed by an Alexa Fluor® 647 secondary antibody. Panels (**C, F, I** and **L**) present the merged images. Slides were mounted and examined under an epifluorescence microscope (Nikon Eclipse 80i, 50x immersion objective).

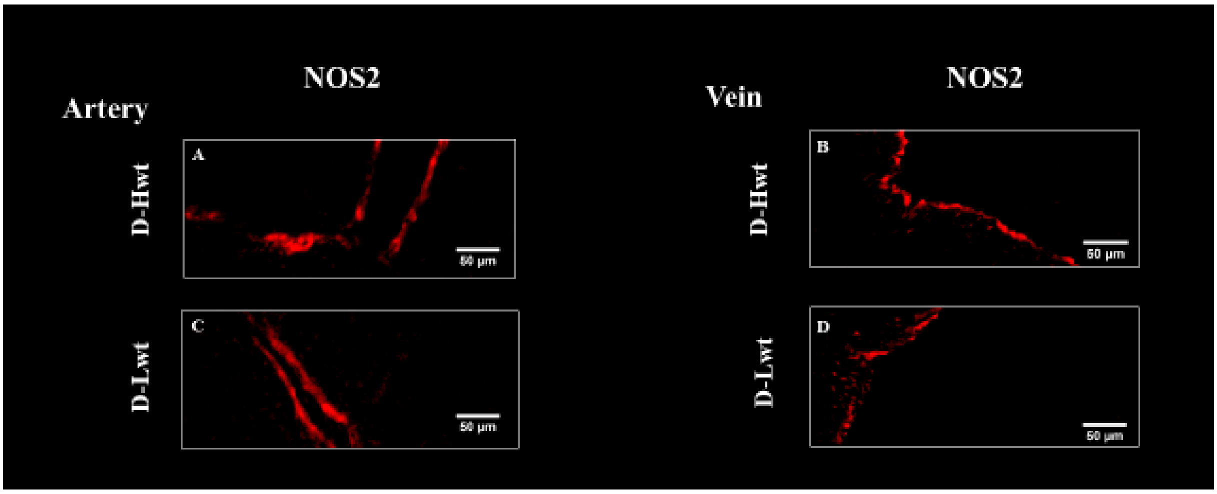

Figure S2. Representative epifluorescent images of the umbilical cord vessels endothelium immunolabelled for inducible nitric oxide synthase (NOS2)

Visualization of the epifluorescent images of the immunolabelled umbilical cord endothelium originated from mature birth weight-discordant Hwt and Lwt siblings. Panels (**A, C**) and (**B, D**) represent the artery and the vein respectively, immunolabelled with anti NOS2 antibody and followed by an Alexa Fluor® 647 secondary antibody. Slides were mounted and examined under an epifluorescence microscope (Nikon Eclipse 80i, 50x immersion objective).

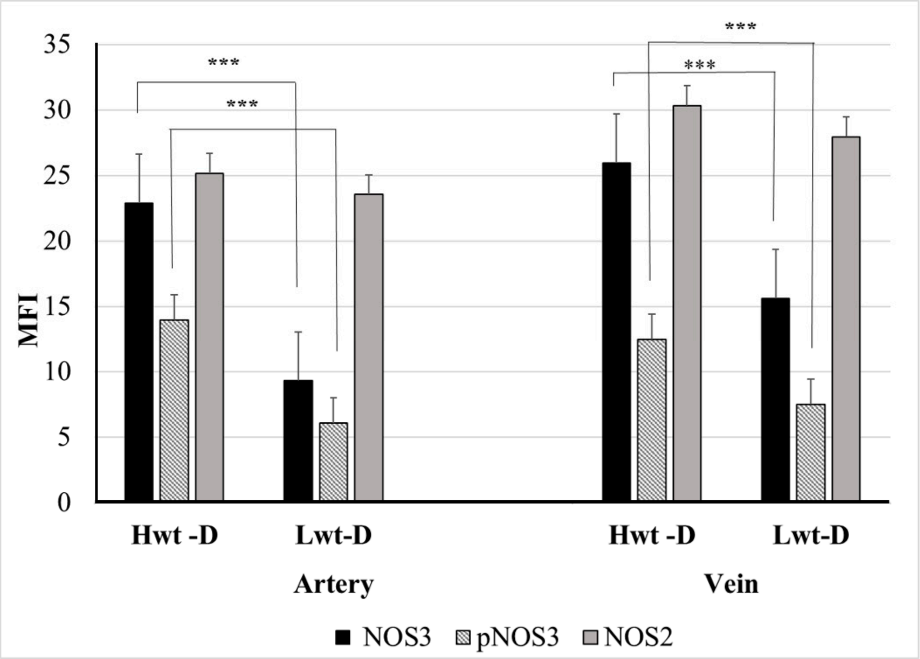

Figure S3. Endothelial (NOS3) and inducible nitric oxide synthase (NOS2) levels in the umbilical cord vessels

Comparison of the mean fluorescence intensity (MFI) values of immunolabelled umbilical cord vessels, originated from high (D-Hwt) and low weight twin neonates (D-Lwt), (n=18 pairs). UCs were stained for NOS3, its phosphorylated status at Ser1177 residue (pNOS3), and NOS2. Statistical significance was accepted at ***$p < 0.001$ based on one-way ANOVA using the Newman-Keuls multiple comparison test.

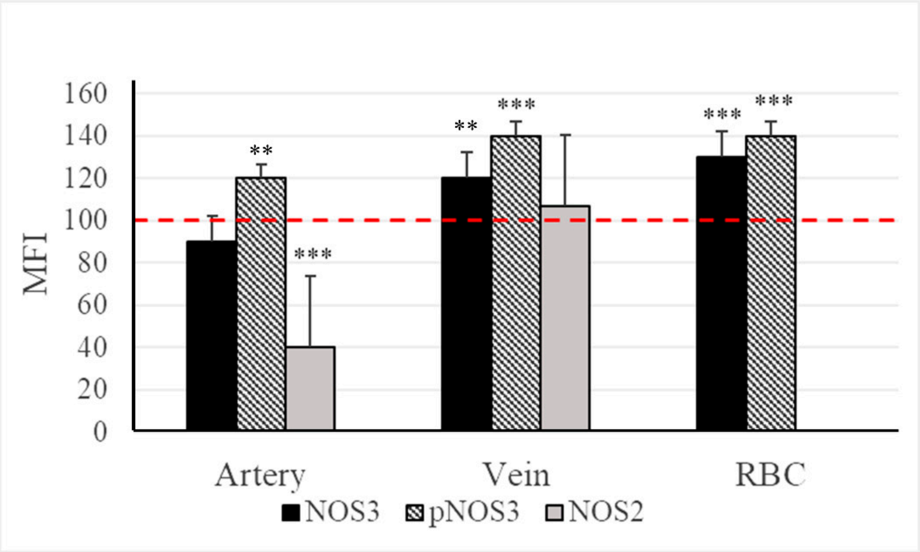

Figure S4. Evaluation of the expression pattern of endothelial (NOS3) and inducible nitric oxide synthases (NOS2) and the phosphorylation status of NOS3 (pNOS3) at the Ser1177 residues in between the mature birth weight-discordant versus non-discordant **high** weight twin groups

Measurement of the mean fluorescence intensity (MFI) were expressed in percentage of NOS3, pNOS3 and NOS2, in the umbilical cord vascular system which includes the arteries, vein and the circulating fetal red blood cell populations (RBCs) of the mature birth weight-discordant twin neonates in comparison to their age and weight matched non-discordant twins, taken as control. The red dotted line represents the non-discordant intensity level at 100%. n=18 pairs. Statistical significance was accepted at **p<0.01 and ***p< 0.001 based on one-way ANOVA using the Newman-Keuls multiple comparison test. Statistical analysis was performed on measured MFI data set. Birth weight-non-discordant data set was published in Antioxidants 2020; doi: 10.3390/antiox9090845

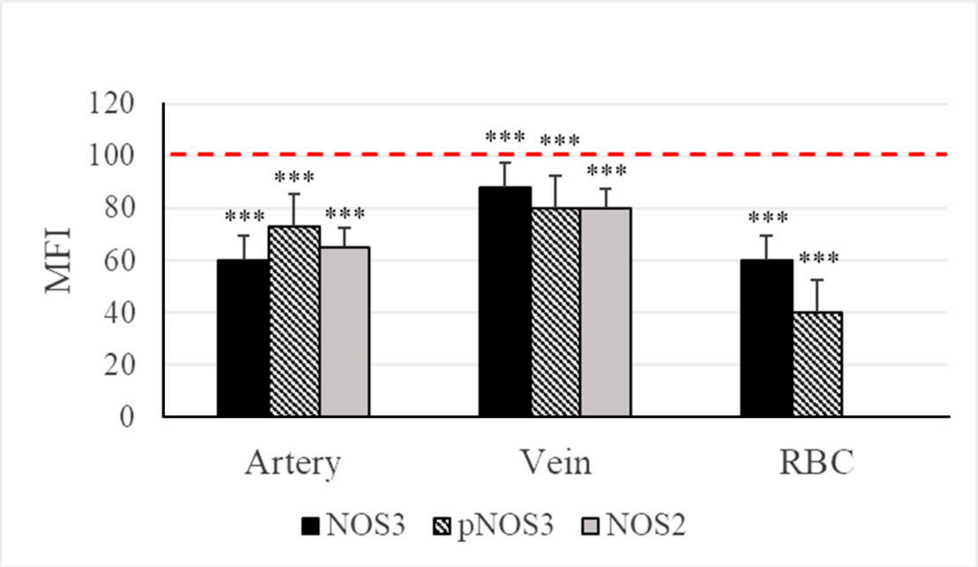

Figure S5. Evaluation of the expression pattern of endothelial (NOS3) and inducible nitric oxide synthases (NOS2) and the phosphorylation status of NOS3 (pNOS3) at the Ser1177 residues in between the mature birth weigh-discordant versus non-discordant **low** weight twin groups

Measurement of the mean fluorescence intensity (MFI) were expressed in percentage of NOS3, pNOS3 and NOS2, in the umbilical cord vessels and red blood cells (RBCs) of the mature birth- weight discordant twin neonates in comparison to their age and weight matched non-discordant twins, taken as control. The red dotted line represents the non-discordant intensity level at 100%. n=18 pairs. Statistical significance was accepted at ***p< 0.001 based on one-way ANOVA using the Newman-Keuls multiple comparison test. Statistical analysis was performed on measured MFI data set. Birth weight-non-discordant data set was published in Antioxidants 2020; doi: 10.3390/antiox9090845.

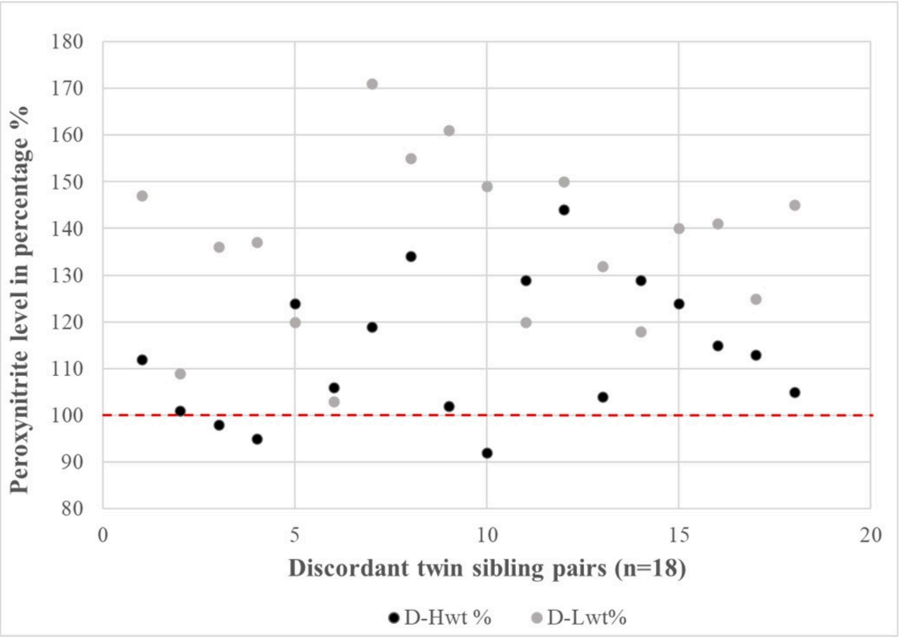

Figure S6. Distribution pattern of the peroxynitrite level between the mature birth weig- discordant twin siblings

Graphical representation indicates the frequency of high and low peroxynitrite level between the mature high weight (D-Hwt, black dots) and low weight (D-Lwt, grey dots) siblings with birth weight-discordancy, where n=18 pairs. The red dotted line in the graph marks 100% intensity level of age and weight matched non-discordant twin neonates.

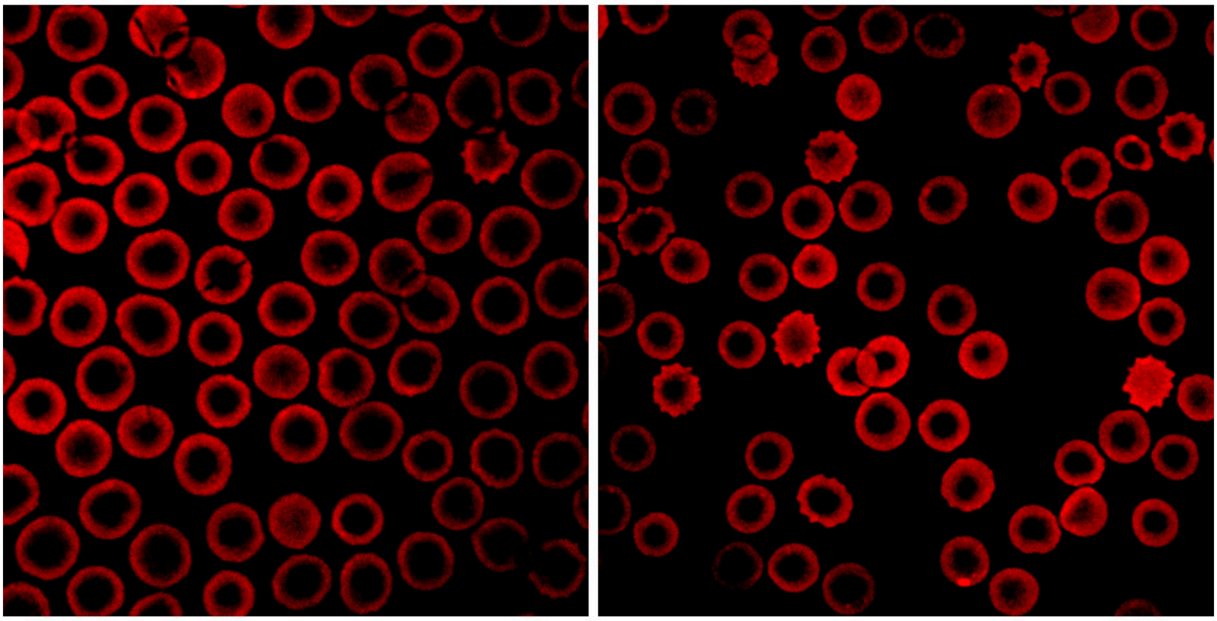

D-Hwt D-Lwt

Figure S7. Representative epifluorescent images of red blood cell variants originated from high (D-Hwt) and low (D-Lwt) birth-weight siblings

Blood smears were immunolabelled for anti-endothelial nitric oxide synthase/Alexa®647-conjugated antibodies, mounted in Antifading, BrightMount/Plus aqueous mounting medium, and examined under epifluorescence microscope (Nikon Eclipse 80i, 100x immersion objective) with a QImaging RETIGA 4000R camera, using Capture Pro 6.0 software.
